# Contribution of Herpesvirus Specific CD8 T Cells to Anti-Viral T Cell Response in Humans

**DOI:** 10.1371/journal.ppat.1001051

**Published:** 2010-08-19

**Authors:** Elena Sandalova, Diletta Laccabue, Carolina Boni, Anthony T. Tan, Katja Fink, Eng Eong Ooi, Robert Chua, Bahar Shafaeddin Schreve, Carlo Ferrari, Antonio Bertoletti

**Affiliations:** 1 Singapore Institute for Clinical Sciences, A*STAR, Singapore; 2 Unit of Infectious Diseases and Hepatology, Azienda Ospedaliero-Universitaria di Parma, Parma, Italy; 3 Singapore Immunology Network, A*STAR, Singapore; 4 Emerging Viral Diseases, Duke – NUS Graduate Medical School, Singapore; University of Massachusetts Medical School, United States of America

## Abstract

Herpesviruses infect most humans. Their infections can be associated with pathological conditions and significant changes in T cell repertoire but evidences of symbiotic effects of herpesvirus latency have never been demonstrated. We tested the hypothesis that HCMV and EBV-specific CD8 T cells contribute to the heterologous anti-viral immune response. Volume of activated/proliferating virus-specific and total CD8 T cells was evaluated in 50 patients with acute viral infections: 20 with HBV, 12 with Dengue, 12 with Influenza, 3 with Adenovirus infection and 3 with fevers of unknown etiology. Virus-specific (EBV, HCMV, Influenza) pentamer+ and total CD8 T cells were analyzed for activation (CD38/HLA-DR), proliferation (Ki-67/Bcl-2_low_) and cytokine production. We observed that all acute viral infections trigger an expansion of activated/proliferating CD8 T cells, which differs in size depending on the infection but is invariably inflated by CD8 T cells specific for persistent herpesviruses (HCMV/EBV). CD8 T cells specific for other non-related non persistent viral infection (i.e. Influenza) were not activated. IL-15, which is produced during acute viral infections, is the likely contributing mechanism driving the selective activation of herpesvirus specific CD8 T cells. In addition we were able to show that herpesvirus specific CD8 T cells displayed an increased ability to produce the anti-viral cytokine interferon-γ during the acute phase of heterologous viral infection. Taken together, these data demonstrated that activated herpesvirus specific CD8 T cells inflate the activated/proliferating CD8 T cells population present during acute viral infections in human and can contribute to the heterologous anti-viral T cell response.

## Author Summary

The majority of humans are infected by herpesviruses, such as Epstein-Barr virus and Human Cytomegalovirus, which rarely cause severe pathology but heavily distort the human T cell repertoire. Up to 20% of cytotoxic T cells can be specific to Epstein-Barr and Cytomegalovirus. It is believed that all these herpesvirus specific T cells are needed to control the persistent infection. However, it has not been explored whether these T cells can contribute to the immune response to a new viral infection. To investigate this possibility, we analyzed the volume of activated virus-specific and total T cells in patients with acute hepatitis B, dengue, influenza and adenovirus infections. We observed that all acute viral infections trigger an expansion of activated T cell population, part of which is specific to infecting agent, and the other part to herpesviruses. Our study provides evidence that persistent herpesvirus infections alter the composition of the T cell population which is activated during new acute viral infection.

## Introduction

Over the course of the human lifetime, we are exposed to and infected by many different organisms which may be eliminated or may persist. The co-existence of microorganisms in humans is mainly perceived to have negative consequences for health and wellbeing, but examples of potential symbiotic relationship between the host and microbes start to be recognized [1], [2].

Classic examples of microorganisms establishing persistent infections in humans are Epstein Barr virus (EBV) and human cytomegalovirus (HCMV) which are both from ubiquitous *herpesviridae* family of viruses which infect more than 90% of the human populations. These viruses are associated with the development of specific tumors (i.e. Burkitt's Lymphoma) and they can reactivate with significant pathological consequences in immunocompromised hosts [3], [4]. Nevertheless, in most of the cases, herpesvirus infections are subclinical and well tolerated, even though they cause a robust distortion of T cell repertoire [5], [6] with HCMV and EBV-specific CD8 T cell known to represent up to 20% of total CD8 T cell population [7], [8], [9]. Our inherent effort to maintain such a large population of virus-specific T cells, is seen as a necessity to suppress CMV and EBV reactivation in humans [9], [10], [11]. This would imply that the sole function of herpesvirus specific memory effector CD8 T cells is to act against CMV and EBV infected cells. However, evidence in animal models have shown that effector or memory CD8 T cells can provide immune protection against infection with unrelated intracellular pathogens through production of Interferon γ (IFN-γ) [12]. Such data open the possibility that the large population of HCMV and EBV-specific CD8 T cells present in humans might contribute to the immunological response against other pathogens.

Thus, we set out to evaluate whether CD8 T cells specific for herpesviruses can contribute to the anti-viral T cell response triggered by heterologous acute viral infection in humans. CD8 T cell responses to acute viral infections were analyzed sequentially (from onset to recovery) by measuring the population of activated/proliferating CD8 T cells in patients with acute Hepatitis B Virus (HBV), influenza, dengue and adenovirus infections. The combination of activation and proliferation markers (CD38, HLA-DR, Ki-67 and Bcl-2) expressed by CD8 T cells have been recently proposed to identify the whole population of virus-specific effector CD8 T cells induced by viral infection [13]. These results were obtained in subjects receiving attenuated virus vaccines (Smallpox and Yellow Fever), and activation (CD38/HLA-DR) and proliferation markers (Ki-67/Bcl-2 low) were only expressed by CD8 T cells specific for the vaccine but not by CD8 T cells of different specificities.

In contrast, we demonstrate here that acute symptomatic viral infections trigger an expansion of activated/proliferating CD8 T cell populations of variable sizes, comprising CD8 T cells specific for the infecting virus but these populations are also invariably inflated by CD8 T cells specific for persistent herpesvirus infections. The increased sensitivity of HCMV and EBV-specific CD8 T cells to IL-15 is the likely explanation of this in vivo observation. In addition, HCMV and EBV specific CD8 T cells demonstrate, at the peak of acute infection, an increased ability to secrete IFN-γ suggesting that they might functionally contribute to the heterologous acute anti-viral immunity.

## Results

### The size of anti-viral CD8 T cell response in acute hepatitis B

We initially evaluated the size and the expansion kinetics of CD8 T cell population during acute hepatitis B infection. The frequency and quantity of CD8 T cells expressing CD38/HLA-DR and Ki-67/Bcl-2 phenotypic markers was analyzed in 20 patients with acute hepatitis B. Samples were collected at multiple time points from onset of disease (HBsAg+, ALT>1000 U/L) to full recovery (HBsAg- at least 1 month after onset).

**Figure 1:**
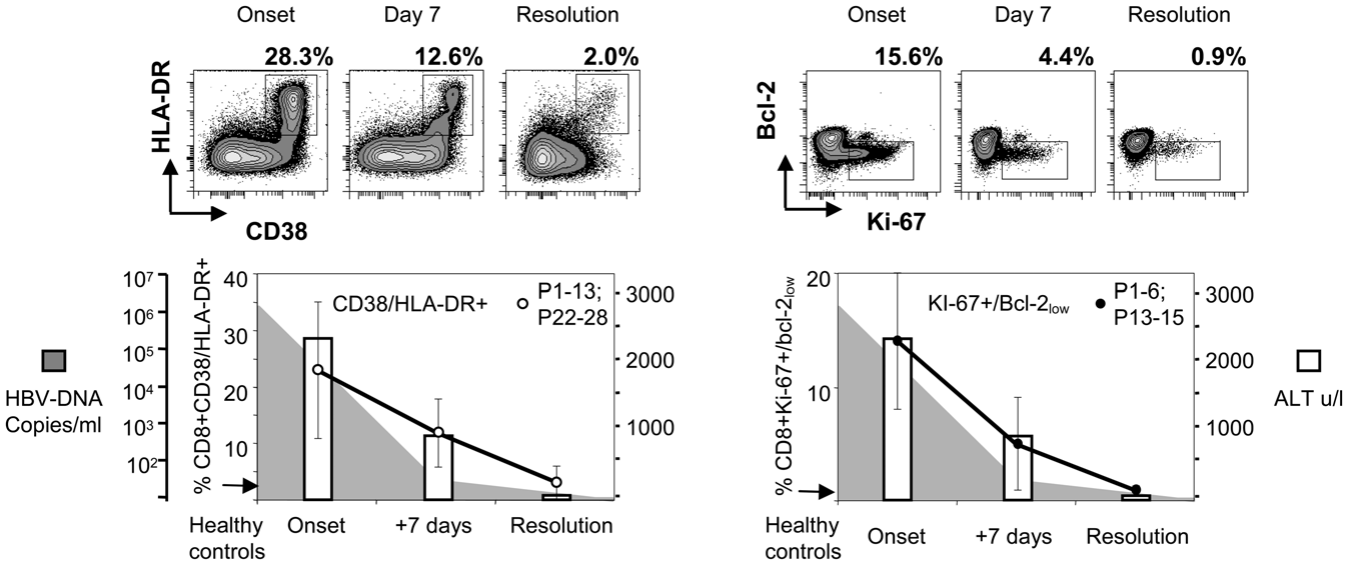
Activated and proliferating CD8 T cells during acute hepatitis B infection. Longitudinal CD38/HLA-DR (left) and Ki-67/Bcl-2 (right) expression on total CD8 T cells in patients with acute HBV infection (n = 20) is shown. Ki-67/Bcl-2 expression was done in 9 out of 20 patients. FACS contour plots were gated on CD3/CD8 positive cells. Percentages of double positive cells are shown. The arrow indicates the level of activation/proliferation found in healthy controls (the average of 5). White bars indicate the mean serum ALT levels, shaded area shows the mean serum HBV DNA of the patients tested.

A remarkably large expansion of activated CD8 T cell pool was detected. CD38/HLA-DR markers were expressed by approximately a quarter of total CD8 T cells (mean 23%, range 12–68%) at the onset of clinical hepatitis. The frequency of CD38/HLA-DR+ CD8 T cells decreased consistently at the second time point (8–10 days later, mean 12%; range 4–22%) and at the time of recovery it returned to the normal level (mean 3%, range 0.9–10%), detectable in healthy controls (Figure 1, left panel). CD8 T cells co-expressing Ki-67 and low Bcl-2 followed identical kinetics. The peak of Ki-67/Bcl-2 low CD8 T cells was detected at the onset of disease (mean 14%, range 4.5–27% of total CD8 T) and contracted abruptly after 10 days (mean 5%, range 0.8–11%) and at the resolution minimal proliferation was detected (mean 0.8%, range 0.4–1.6%) (Figure 1, right panel).

### Antigen-specificity of the activated/proliferating CD8 T cell population

**Figure 2:**
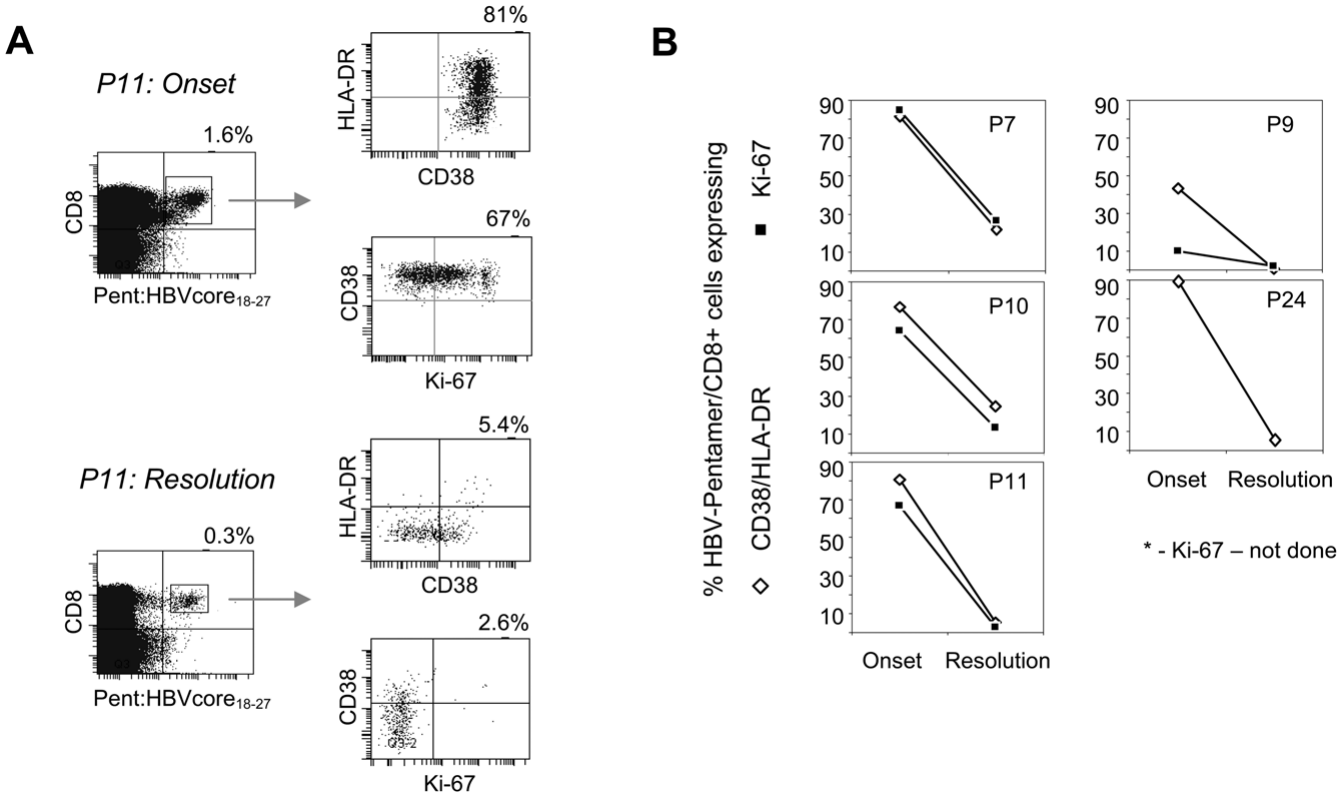
Activation and proliferation of HBV-specific T cells during acute HBV infection. A) CD38/HLA-DR and Ki-67 expression on HBV core18–27 pentamer + CD8 T cells at the onset of disease and after resolution (+35 days) in a representative patient. B) Percentages of HBV-pentamer + CD8 T cells expressing CD38/HLA-DR and Ki-67 in 5 patients over time.

To analyze whether the population of activated/proliferating CD8 T cells included HBV-specific CD8 T cells, HBV-specific pentamers were used to directly visualize these cells in 5 HLA-A201+ patients. Figure 2 A and B shows that the expression of activation markers of HBV-specific CD8 T cells followed the kinetics of expression of the total CD8 T cell population. HBV-pentamer+ CD8 T cells expressed activation markers and proliferated at the onset of disease but not at the recovery phase (Figure 2 A and B). These results demonstrate that HBV-specific CD8 T cells are represented within the total population of activated/proliferating total CD8 T cells.

Then we tested whether CD8 T cell specific for other common viruses (CMV, EBV, influenza) quantitatively contribute to the total pool of activated/proliferating CD8 T cells. A set of HCMV, EBV or Influenza pentamers (Supplementary Table S1) was used to detect CD8 T cells specific for these common infections. We visualized a sizeable ex vivo frequency of HCMV, Influenza and EBV-specific CD8 T cells in 13 acute hepatitis B patients and their expression of CD38/HLA-DR and Ki-67 was tested at the onset of acute hepatitis and after recovery.

**Figure 3:**
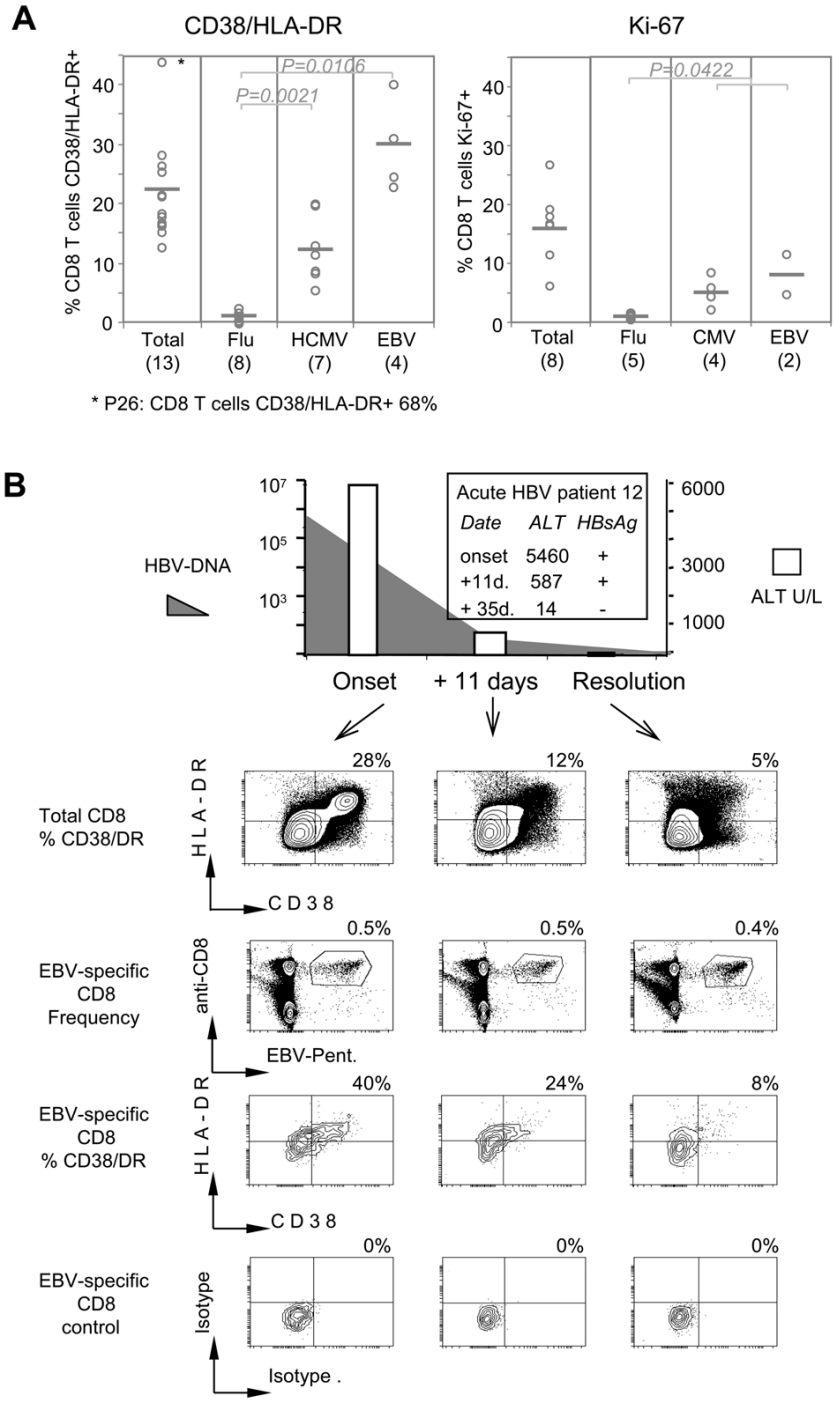
Activation of HCMV and EBV-specific CD8 T cells during acute hepatitis B infection. A) Frequencies of total, and Influenza, HCMV, EBV specific CD8 T cells expressing CD38/HLA-DR and Ki-67 at the onset of acute hepatitis B. Data shown only for the patients where virus-specific CD8 T cells were visualized (P7, P9, P10–13, P22–28). The numbers in brackets indicate the number of patients tested. P value was calculated using Mann Whitney test. The differences are considered significant when the P value <0.05. B) Phenotypic profile of total CD8 T and EBV-specific (BZLF-1 190–197+ EBNA-3A 193–201) CD8 T cells over time in a representative acute hepatitis B patient (P 12). Percentages of respective gates/upper left quadrants are shown.

A remarkably different profile of CD8 T cell activation was detected in relation to the CD8 T cell specificity. While influenza-specific CD8 T cells were neither activated (8 out of 8 patients) nor proliferating (5 out of 5 tested patients) at all time points (Figure 3 A and Supplementary Figure S1), HCMV and EBV-specific CD8 T cells were activated (HCMV mean 12.5%; EBV mean 30%) and proliferating (HCMV mean 4.9%; EBV mean 8%) (Tables 1–2 and Figure 3 A) in all the acute HBV patients where such cells were detectable. The expression of CD38/HLA-DR and Ki-67 markers in HCMV and EBV-specific CD8 T cells followed the same expression kinetics of total and HBV-specific CD8 T cells and contracted after recovery as shown on Figure 3 B (patient 12).

**Table 1:** Activated HBV-, HCMV-, EBV- and Flu-specific CD8 T cells in HBV acute patients where HBV, HCMV, EBV and Influenza specific cells could be visualized with pentamers.

Patients (HBV Acute)	CD38+/HLA-DR+CD8+ T cells % (absolute number/10 µl)				
	Total CD8	HBV	HCMV	EBV	FLU
P 7	18% (738)	81% (108)	5.5% (2)	n f	n f
P 9	18% (720)	43% (14)	13% (10)	n f	n f
P 10	25% (940)	77% (9)	20% (20)	n f	0% (0)
P 11	21% (800)	81% (54)	n f	n f	0.9% (0)
P 12	28% (1218)	n f	8% (9)	41% (12)	n f
P 13	17% (700)	n f	n f	31%	n f
P 22	17% (700)	n f	n f	n f	2% (0)
P 23	15% (880)	n f	12%	n f	1.3% (0)
P 24	26% (1066)	89%	20%	n f	1.4% (0)
P 25	12% (710)	n f	9% (10)	26% (9)	0% (0)
P 26	68% (n.d.)	n f	n f	22% (n d)	n f
P 27	16% (n d)	n f	n f	n f	0.2% (n d)
P 28	21% (n d)	n f	n f	n f	0% (n d)
Average	23%	74%	12.5%	30%	0.7%

Percent of activated virus-specific CD8 T cells are shown and the number of cells in 10 µl of blood, calculated based on the pentamer frequency and lymphocyte count in the blood of patients at the acute stage.

n f  =  not found; n d  =  not done.

**Table 2:** Proliferating HBV-, HCMV-, EBV- and Flu-specific CD8 T cells in HBV acute patients where HBV, HCMV, EBV and Influenza specific cells could be visualized with pentamers.

Patients (HBV Acute)	Ki-67+ CD8+ T cells % (absolute number/10 µl)				
	Total CD8	HBV	HCMV	EBV	FLU
P 7	19% (738)	85% (112)	2% (1)	n f	n f
P 9	6% (240)	10% (6)	2.4% (2)	n f	n f
P 10	16% (564)	65% (8)	6% (6)	n f	0% (0)
P 11	17% (560)	67% (40)	n f	n f	0.1% (0)
P 12	n d	n f	n d	n d	n f
P 13	16% (739)	n f	9% (12)	11% (1)	n f
P 22	n d	n f	n f	n f	n d
P 23	n d	n f	n d	n f	n f
P 24	n d	n d	n d	n f	n d
P 25	12% (520)	n f	n d	5% (0.2)	0% (0)
P 26	n d	n f	n f	n d	n f
P 27	18% (n d)	n f	n f	n f	2.5% (n d)
P 28	27% (n d)	n f	n f	n f	0% (n d)
Average	16%	57%	4.9%	8%	0%

Percent of proliferating virus-specific CD8 T cells are shown and the number of cells in 10 µl of blood, calculated based on the pentamer frequency and lymphocyte count in the blood of patients at the acute stage.

n f  =  not found; n d  =  not done.

The differential phenotype of CD8 T cells specific for different viruses during acute hepatitis B was well represented in a patient (patient 10, Supplementary Figure S1) where the different CD8 T cells specificities co-exist in different activation states. As already shown in Figure 2, at the peak of acute hepatitis, HBV-specific CD8 T cells are mostly activated (77%) and proliferating (65%). At the same time point, a proportion of HCMV-specific CD8 T cells are also expressing activation (20%) and proliferation (6%) markers, while influenza-specific are in a complete resting phenotype. Taken together, these data demonstrated that, at least in acute hepatitis B infection, a sizable proportion of CD8 T cells specific for persistent viruses are activated during acute heterologous infection.

### Activated HCMV/EBV specific CD8 T cells during acute viral infections

Evidence of activation of unrelated virus specific CD8 T cells has been also reported in HIV infection [14], but our results clearly differ from the ones obtained in attenuated virus vaccine recipients [13], where activation of Influenza, HCMV and EBV was not reported. Thus we tested whether our observation was peculiar to acute HBV infection or whether it represents a common feature in other acute viral infections in human.

**Figure 4:**
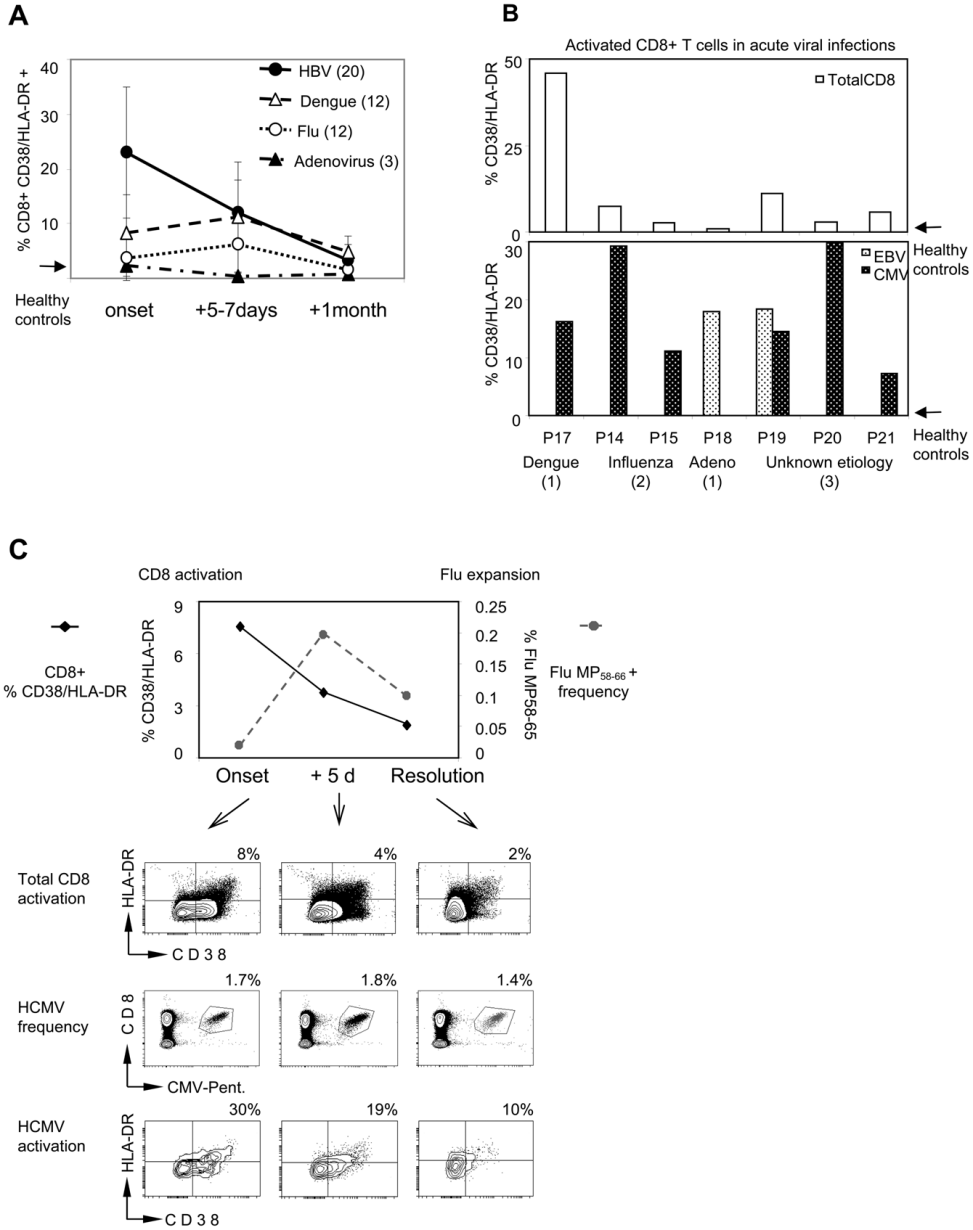
Activation of CD8 T cells in different acute viral infections. A) Kinetics of the expansion of CD38/HLA-DR expressing CD8 T cells in acute HBV (n = 20), dengue (n = 12), influenza A (n = 12), adenovirus (n = 3) infected patients. B) Frequencies of total CD8 T (upper panel) and HCMV and EBV-specific CD8 T cells (lower panel) expressing CD38/HLA-DR at the onset of acute viral infections. The number of patients in whom HCMV and EBV CD8 T cells were sizeable is indicated by digits. The arrow indicates activation levels of healthy controls (n = 5). C) Discordant kinetics of activated total CD8 T cells and influenza-A specific CD8 T cell frequency in one patient (P14). Contour plots represent the phenotype of total CD8 T cells (upper panel) and of HCMV-specific (pp65, 495–504) CD8 T cells (lower panel). The middle panel displays the frequencies of HCMV-pentamer+ CD8 T cells.

Samples from patients with acute Dengue (n 12), Influenza A (n 12), Adenovirus (n 3) infections were collected at the onset of disease (represented in these patients by fever >38° C), after 5–7 days and after recovery (∼21 days) and frequency of CD8 T cell population expressing activation (CD38/HLA-DR) and proliferation (Ki-67) markers was measured (Figure 4 A). Differences in the magnitude and kinetics among diseases with different etiology were found. Adenoviral infection elicited a minimal activation of CD8 T cell population (mean 3.5%), which is only slightly higher than that of healthy individuals (mean of 5 healthy controls 2.4%). In addition, the peak frequency of activated total CD8 T cells in dengue and influenza infections is detected 5–7 days after onset of fever unlike that of HBV, where the peak frequency is seen at the onset of disease (Figure 4 A). These different profiles are compatible with the fact that the onset of disease in acute hepatitis (jaundice) is represented by liver injury and coincides with the peak of adaptive immune response [15], [16] while dengue and influenza infections trigger a strong innate immune reaction (febrile status being a clinical manifestation) and thus, in these infections, full maturation of virus-specific adaptive immunity is expected to peak ∼5–7days after infection.

Nevertheless, despite the lower quantity of total activated CD8 T cells in dengue, influenza, adenovirus patients as well as in 3 subjects with fever of unidentified etiology, the CD38/HLA-DR expression profile on HCMV or EBV specific CD8 T cells was similar to that in acute HBV infection. Figure 4 B summarizes the results obtained in the patients with the indicated pathologies, where a sizeable ex vivo frequency of HCMV or EBV specific CD8 T cells was detected. HCMV and EBV specific CD8 T cells (lower panel) express activation markers to a level even higher to what is detected in the global CD8 T cells populations (upper panel). Unfortunately, the paucity of the cells obtained in these patients didn't allow us to analyze also Ki-67 expression on HCMV and EBV specific CD8 T cells, but overall these results demonstrate that activation of CD8 T cells specific for persistent viral infection (HCMV-EBV) is a constitutive feature of acute anti-viral immunity in human.

Interestingly, we were able to study a patient with acute influenza infection in whom, influenza-specific CD8 T cell expansion didn't coincide with the profile of total activated CD8 T cells (Figure 4 C, P14). In this subject, the influenza-specific CD8 T cells (specific for matrix protein 58–66 epitope) could be visualized only 5 days after onset of symptoms. In contrast, HCMV-specific (pp65 123–131) CD8 T cell frequency was comparatively constant at different time points (1.7% onset; 1.8% +5 days; 1.4% +14 days), and already co-expressed activation markers (30%) at the onset of disease (Figure 4 C). Thus, in this patient, the whole CD38/HLA-DR+ population before the expansion of the CD8 T cells specific for the acutely infected virus seems to be composed of herpesvirus-specific activated CD8 T cells.

### Selective activation of HCMV/EBV-specific CD8 T cells by IL-15

We investigated the possible mechanisms of the selected activation of HCMV and EBV- specific CD8 T cells in patients with heterologous acute viral infections.

CD8 T cell cross-reactivity, reactivation of the HCMV or EBV infection and/or activation mediated by cytokines can be implicated in this phenomenon.

Cross-reactivity between HCMV or EBV-specific CD8 T cells with epitopes present in the acute heterologous virus infection seems unlikely. The cross-reactive potential of HCMV-specific CD8 T cells is very uncommon [7] and our data do not support cross-reactive mechanisms either. We could detect activation of CD8 T cells specific for two distinct immediate early and latent EBV epitopes (HLA-B8 RAKFKQLL, BZLF-1 190–197, and HLA-B8 FLRGRAYGL, EBNA-3A 193–201) in an acute HBV patient (Supplementary Figure S2). If cross-reactivity was responsible of this activation, it would require that both epitopes share sequence or structural similarity with HBV virus, an unlikely scenario, based on a sequence similarity search (NCBI PubMed BLAST), which demonstrated no sequence overlap (lowest E value obtained  = 11) between these EBV epitopes and HBV proteome.

Reactivation of HCMV and EBV could be a plausible cause, and it might explain why CD8 T cells specific for Influenza are not activated in acute HBV infections. To investigate this possibility, HCMV and EBV DNA levels were tested longitudinally in the serum. However, we did not find any evidence of HCMV or EBV reactivations. HCMV-DNA and EBV-DNA titers were below the level of detection in all patients (HBV, Dengue, Influenza, Adenovirus and fever of unidentified etiology) from the onset of acute heterologous viral infections to recovery (data not shown). Importantly, although HCMV and EBV reactivations are usually associated with the expansion of HCMV/EBV- specific CD8 T cells [10], [17], [18] significant changes in the EBV or HCMV specific T cells quantity were not observed through the course of acute infections (Figure 3 B and Supplementary Figure S2).

We therefore analyzed whether cytokines produced during acute viral infections [19] can be responsible for the differential expression of activation markers by EBV-, HCMV- and influenza-specific CD8 T cells.

**Figure 5:**
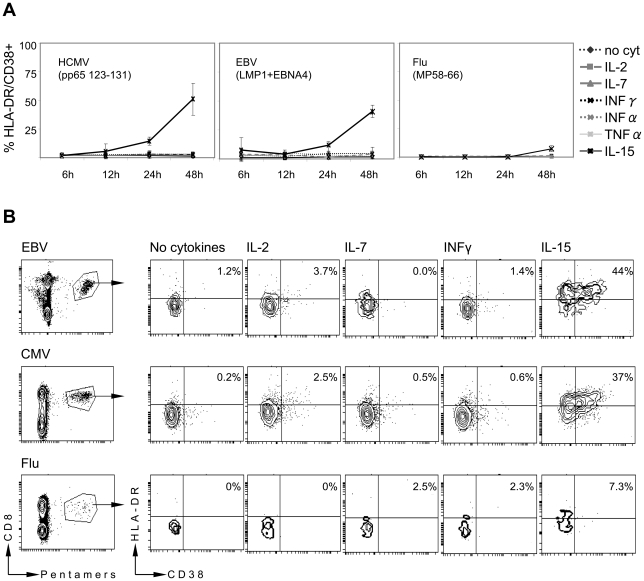
IL-15 induced activation of HCMV and EBV-specific CD8 T cells. A) PBMC of 4 healthy volunteers were incubated with IL-15 (10 ng/ml), IL-2 (20 U/ml), IL-7 (10 ng/ml), IFN-γ (100 U/ml), IFN- α (100 U/ml), TNF- α (100 U/ml). Cells were harvested at the indicated time points and frequency of CD38/HLA-DR expressing virus specific CD8 T cells was analyzed. B) Contour plots show the CD38/HLA-DR expression of EBV, HCMV or Influenza specific CD8 T cells after 48 hours of incubation with the indicated cytokines in a representative healthy subject.

PBMC or purified CD8 T cells of healthy subjects containing resting EBV, HCMV and Influenza specific CD8 T cells were incubated with different concentrations of IL-15, IL-2, IL-7, IFN-γ, IFN-α and TNF-α and the expression of HLA-DR and CD38 on EBV, HCMV and Influenza specific CD8 T was analyzed at different intervals (Figure 5 A). We detected that after 24 and 48 hours of incubation, IL-15 (at 1 and 10 ng/ml) induced CD38/HLA-DR expression in HCMV and EBV specific CD8 T cells while the other inflammatory cytokines did not activate EBV and HCMV specific CD8 T cells. Similar to the in vivo findings, influenza-specific CD8 T cells were not or only weakly activated by addition of any of the tested cytokines (Figure 5 A and B). Prolonged incubation times (3 to 5 days) did not alter the activation profile. Figure 5 B shows the results obtained in one healthy subject where HCMV, EBV and Influenza specific CD8 T cells were simultaneously detected. Incubation of total PBMC with IL-15 induces expression of CD38/HLA-DR molecules in EBV and HCMV specific CD8 T cells (44% and 37% respectively) but only in few influenza-specific CD8 T cells (7%). The specific effect of IL-15 on HCMV and EBV-specific CD8 T cells was confirmed in other healthy subjects where individual specificities were detected (HCMV =  n4; EBV =  n4). Similar results were obtained incubating total PBMC or CD3+ CD8+ purified cells (not shown). Thus, IL-15, a cytokine that has been shown to induce T cell activation in mice [20] and human [21], [22] and is known to be produced during acute viral infections ([19] and personal data) induces preferential CD38, HLA-DR up-regulation of HCMV and EBV-specific CD8 T cells rather than influenza-specific ones.

### Functional analysis of HCMV and EBV-specific CD8 T cells during acute heterologous viral infections

Having observed that a proportion of HCMV and EBV-specific CD8 T cells are activated during heterologous acute viral infection, we sought to analyze their functional profile. The limited quantity of cells available in patients with acute viral infections precludes an extensive evaluation of the functional profile directly in our patient sample. Thus, since IL-15 mimics the differential activation state of HCMV, EBV and influenza-specific CD8 detected in patients with acute viral infections, we performed a series of functional experiments using PBMC of healthy individuals activated with IL-15.

**Figure 6:**
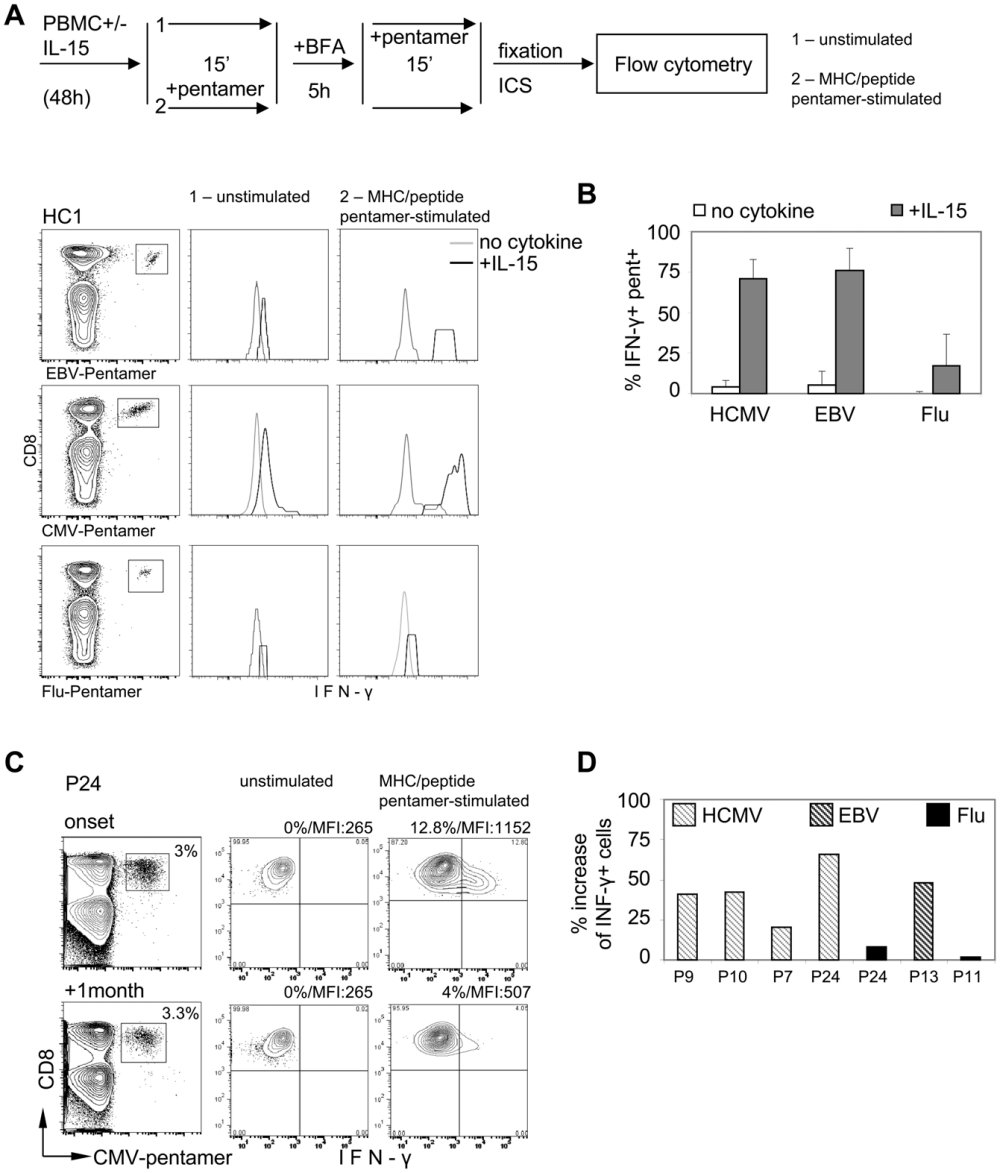
Functional analysis of HCMV and EBV-specific CD8 T cells during acute heterologous viral infections. A) Schematic representation of experimental design: PBMC from healthy volunteers were incubated for 48 h in presence or absence of IL-15. Cells were then collected, incubated with pentamer or in PBS for 15 min, washed and incubated for 5 h in presence of Brefeldin A. Following the 5 h incubation the cells were collected and the unstimulated cells, those that have not been stained with pentamer prior to BFA incubation, were stained with pentamer for 15 min. Then the cells were washed and ICS performed. The expression of IFN-γ in EBV-pentamer+ (EBNA-1 407–417, +EBNA4, 416–424), HCMV-pentamer+ (pp65 495–504) and Flu-pentamer+ (MP, 58–66) CD8 T cells is shown. Histogram plots gated on CD3+ CD8+ pentamer + cells (contour plots) of one representative healthy individual are shown. B) Percent of IFN-γ+ MHC/peptide pentamer stimulated HCMV-, EBV- and Flu-specific CD8 T cells is shown based on gating for unstimulated cells cultured without cytokine. C) Histogram plots representing IFN-γ production of unstimulated and MHC/peptide pentamer stimulated HCMV-pentamer+ (pp65 495–504 + pp65 123–131) CD8 T cells in a representative HBV acute patient. D) Percent increase of IFN-γ production by MHC-peptide pentamer stimulated HCMV-, EBV- and Flu-specific CD8 T cells during the onset of acute HBV infection compared to the resolution.

We first tested whether IL-15 can differentially trigger T cell activation in HCMV, EBV and Influenza specific CD8 T cells *in vitro*. PBMC of healthy individuals were incubated with or without IL-15 for 48 hours and HCMV, EBV and influenza-specific CD8 T cells were tested for their ability to produce anti-viral cytokines (IFN-gamma, IL-2 and TNF-alpha) using intracellular cytokine staining. Note that the cytokines measurement on CD8 T cells specific for the different viruses requires their visualization with the specific HLA-class I/peptides pentameric complex (pentamers). The pentamer staining can potentially trigger T cell stimulation through direct interaction of the TCR with the synthetic MHC-class I peptide complexes of the pentamers[23], [24], [25]. Thus, to distinguish whether IL-15 can directly trigger T cell activation (TCR-independent stimulation) or enhance the T cell activation triggered by MHC/peptide pentamer (TCR-dependent stimulation), the intracellular production of IFN-gamma on CD8 T cells was analyzed adding the MHC/peptide pentamers either before (TCR-dependent stimulation) or after (TCR-independent stimulation) the incubation time of intracellular cytokine staining. A schematic representation of the experimental design is presented in Figure 6 A.

In accordance with previous studies [21], [22], IL-15 elicited a spontaneous production of IFN-gamma on T cells. However, the level of IFN-gamma production was modest and present in CD8 cells irrespective of their specificity. Dot plots displayed in Figure 6 A illustrate these results obtained in one representative subject. Increased production of other cytokines (IL-2, TNF-alfa) was less striking (not shown).

In contrast, we observed that HCMV and EBV-specific CD8 T cells incubated with IL-15 and stained with MHC/peptide pentamer at the beginning of the intracellular cytokine assay showed an increased ability to produce IFN-gamma. More than 70% of IL-15 pulsed HCMV and EBV-specific CD8 T cells produced high quantity of IFN-gamma while in the absence of IL-15, MHC-pentamer staining stimulate only a minority of HCMV and EBV-specific CD8 cells (Figure 6 A–B). Importantly, IL-15 incubation has a modest effect on Influenza specific CD8 T cells (Figure 6 A–B). Thus, our in vitro experiments showed that IL-15 is not only able to preferentially activate HCMV and EBV-specific CD8 T cells, but can also modulate their functional responsiveness to the TCR-dependent stimulation mediated by MHC-pentamer staining.

Having defined a different functional profile on *in vitro* activated HCMV and EBV-specific CD8 T cells, we tested whether such features could be detected *in vivo*.

In line with the experiments *in vitro*, MHC-peptide pentamer stimulation was detected preferentially on HCMV and EBV-specific CD8 cells present during the acute phase of HBV infection (Figure 6 C and D).

Figure 6 C shows the results obtained in a representative patient (P24) with acute hepatitis and with a sizeable population of activated HCMV-specific CD8 T cells (20%). While the spontaneous production of IFN-gamma was identical in HCMV-specific CD8 cells present at the onset and at recovery of acute hepatitis B (Figure 6 C- unstimulated), 12% of HCMV specific CD8 cells present at the onset of acute hepatitis B against only 4% of the ones present at recovery produced IFN-gamma after MHC-pentamer stimulation (Figure 6 C). In addition to the higher frequency of IFN-gamma producing cells, the amount of the cytokine produced during the onset was higher than that during the resolution, as visualized by the difference in mean fluorescence intensity (MFI) (1152 at onset and 507 resolution).

Figure 6 B shows the cumulative results obtained in 6 subjects with detectable HCMV (P9, P10, P7, P24), EBV- (P13) and influenza-specific CD8 (P24, P11) at the onset and recovery of acute hepatitis B. Bars indicate the % increase of IFN-gamma producing CD8 T cells at onset of acute hepatitis in comparison with recovery.

Thus, persistent virus specific CD8 T cells produce more anti-viral cytokines after TCR-mediated activation during acute phase of heterologous viral infection.

## Discussion

We demonstrate here that activation of CD8 T cells specific for persistent viral infection (HCMV-EBV) is a constitutive feature of acute anti-viral immunity in human.

Our conclusions differ from the ones obtained in attenuated virus recipients [13], which have suggested that activated (CD38/HLA-DR+) and proliferating (Ki-67+) CD8 T cells are exclusively constituted of CD8 T cells specific for the acutely infecting virus. However, our patients with activated/proliferating HCMV and EBV responses had a symptomatic viral infection with a high level of inflammation, whereas those subjects vaccinated with attenuated viruses, by definition, should not exhibit any pathology of acute infection.

Of note, the presence of activated HCMV and EBV specific CD8 was also detected during other pathological human viral infections [14], [26], [27] further supporting our conclusion that activation of CD8 T cells specific for persistent infection is a consistent phenomenon during symptomatic viral infections.

In contrast to HCMV and EBV-specific CD8 cells, we observed that CD8 T cells specific for influenza were not activated during the acute phase of heterologous acute viral infection. Thus, our data show that memory CD8 cells specific for persistent and non-persistent viruses not only differs in term of phenotypic profile in healthy individuals [28], but respond differently to the pathological condition triggered by an heterologous acute viral infection.

We can only speculate about the causes of the variable behavior of CD8 T cells specific for the different pathogens. A plausible explanation is that, while influenza-specific CD8 T cells are true memory CD8 cells without any recent encounter to their specific ligand, EBV and HCMV specific CD8 cells might experience a continuous or repetitive exposure to the specific antigens.

The accumulation over time of herpesvirus-specific CD8 T cells in healthy subjects [29], [30] and work in animal model of HCMV infection [31], have suggested that EBV and HCMV antigens are constantly available for T cell stimulation. The Ag-exposure might modulate the functional state of HCMV and EBV-specific CD8 cells and program them to respond to cytokines produced during acute viral infections. The differential functional state of herpesvirus specific CD8 T cells when compared with influenza-specific was confirmed by our *in vitro* data. We clearly demonstrate that IL-15 triggers *in vitro* the activation/proliferation of HCMV, EBV specific rather than influenza-specific CD8 T cells.

Based on these *in vitro* data, we favor the idea that the detection of activated/proliferating HCMV and EBV specific CD8 T cell is mediated principally by the presence of IL-15 during acute phase of viral infections. This makes HCMV/EBV reactivation or indeed cross-reactivity a less likely explanation for this phenomenon. However, it is important to stress that this causative link is hypothetical since the level of IL-15 required to activate HCMV/EBV *in vitro* (1–10 ng/ml) is higher than what we detected in the serum of the patients in this study (always lower then 50 pg/ml in any viral infection, data not shown). Such inconsistency should be taken into account, even though the serum cytokine levels cannot define their actual concentrations in the target organ or lymph node.

We cannot exclude that a reactivation of EBV/HCMV infection is occurring in our patient population and thus directly driving the HCMV or EBV-specific CD8 T cell activation. We couldn't demonstrate any virological evidence of HCMV and EBV reactivation, but the negative virological tests do not exclude a HCMV and/or EBV viral reactivation is present elsewhere outside the blood compartment and is immediately curtailed by activated HCMV and EBV specific CD8 T cells. A similar scenario was suggested to occur in patients with acute Hantavirus infection where an increased EBV-DNA titers were found only in subjects without measurable EBV-specific T cell response [26]. However, it has been reported that HCMV and EBV reactivation is associated with the expansion of HCMV/EBV specific CD8 T cells [10], [17], [18], which was not observed in any of our patients (Figure 3 B and Supplementary Figure S2).

What appears clear from our data is that the contribution of the activated/proliferating HCMV/EBV specific CD8 T to the size of activated total CD8 T cells is not negligible, but at the contrary can alter the quantitative measurement of anti-viral CD8 T response during acute viral infections. A mean of, respectively, 30% and 12.5% of EBV and HCMV-specific CD8 T cells express activation markers during the acute phase of different viral infections and since the combined population of both HCMV-EBV specific CD8 T cells might exceed 20% of total CD8 T cells [7], [9] it is plausible to conclude that EBV/HCMV-specific CD8 T cells can inflate the number of total activated CD8 T cells.

The presence of activated/proliferating CD8 T cells specific for HCMV and EBV during the early phases of different acute viral infection raises several questions. First, it will be interesting to evaluate whether CD8 T cells specific for other persistent viruses (i.e. HSV1 and 2) can actually behave like HCMV or EBV specific CD8 T cells and thus further contribute to the anti-viral CD8 T cell acute response.

A further question might address the biological significance of the herpesvirus specific CD8 T cell activation during heterologous acute viral infections.

There is a possibility that the activation/proliferation state of HCMV/EBV specific CD8 T cells counteracts the potential attrition exerted by the expansion of CD8 T cells specific for the acutely infecting virus [32] and therefore might be important for preventing the reactivation of HCMV/EBV infection. In this regard, our data differ from previous reports in acute HBV infected patients [33], since we did not observe any significant loss of HCMV or EBV specific CD8 T cells. On the contrary, HCMV and EBV-specific CD8 T cell frequency was remarkably constant during the different phases of acute heterologous viral infections and the observed mild proliferation of HCMV and EBV specific CD8 T cells (Figure 3 A and Supplementary Figure S2) might represent a compensatory mechanism counteracting the attrition exerted by the expansion of CD8 T cell specific for the acutely infected virus [30], [34].

In addition, the observation that activation of HCMV and EBV specific CD8 T cells present during the acute phase of heterologous viral infections is associated with a functional increase in the MHC-pentamer mediated CD8 T cell activation further supports the idea that such events might have a broader biological significance.

We can only speculate about the physiological significance of the increased MHC-pentamer mediated CD8 T cell activation. However, a plausible interpretation is that the HCMV and EBV-specific CD8 cells during acute heterologous viral infection are less dependent to possible co-stimulatory effect mediated by additional molecules provided by their target during T cell recognition. Alternatively, the increased response to pentamer-mediated staining might indicate a lower requirement of MHC-class I complexes necessary for T cell activation [24]. These various possibilities will need further investigation, but what our data clearly demonstrate is that functional differences in the ability to produce IFN-gamma are present in different phases of heterologous acute viral infection.

The increased likelihood of activated HCMV, EBV specific CD8 T cells to produce antiviral cytokines after recognition of HCMV and EBV antigens might be beneficial not only in the control of HCMV/EBV reactivation but can actively contribute to the global anti-viral immune response. Evidences in animal model have already shown that T cell activation of non-antigen specific T cells can contribute to the early response against pathogens [12], [35].

On the other hand, the detected hyper-responsiveness of HCMV and EBV- specific CD8 T cells can have an impact on immunopathogenesis of the viral infections [36]. Heterologous immunity have been observed to alter pathogenesis of different viral diseases [37], [38].

In conclusion, we show that the CD8 T cell population activated during acute viral infection is not constituted exclusively by CD8 T cells specific for the newly infected virus. On the contrary, this population is inflated by the presence of activated T cells specific for herpesvirus, directly demonstrating the ability of persistent virus infections to leave a functional imprint on the acute anti-viral T cell response in humans with functional consequences that will require further elucidation.

## Methods

### Patients and samples

Samples were taken from patients or healthy volunteers attending clinics in Singapore (Dengue, Influenza, Adenovirus infections, fevers of unidentified etiology and healthy volunteers) and Italy (HBV infection). Local Review board and Ethical Committees approved the study. Total number of patients is 50: HBV 20, Influenza 12, Dengue 12, Adenovirus 3, patients with fevers of unidentified etiology 3. Number of healthy volunteers enrolled is 5. Age of the subjects ranged from 20 to 54 years old. Patients were selected on the basis of fever >38°C (Dengue, Influenza, Adenovirus) or jaundice (HBV). Diagnosis of dengue (detection of dengue virus by PCR), influenza A (+ isolation of influenza A from nasal swab), adenovirus (isolation of the virus from nasal swab), and HBV (HBsAg +, anti-HBc IgM+ and HBV-DNA+) was performed within 5 days from selection. Acute hepatitis B patients were all HBsAg+ and had ALT>1000 U/L, at the disease onset. All healthy volunteers were asymptomatic. Peripheral blood mononuclear cell isolation from whole heparinized or EDTA blood with Ficoll-Hypaque was performed within 4 hours of drawing. PBMC were analyzed immediately or frozen for subsequent analysis.

### Virological measurements

HBV patients: HBsAg, HBeAg, anti-HBs, anti-HBc IgG and IgM, anti-HBe, anti-HDV, anti-HCV, anti-HIV-1 and -2 were determined by commercial enzyme immunoassay kits (Abbott Labs, IL, USA; Ortho Clinical Diagnostic, Johnson & Johnson, DiaSorin, Vercelli, Italy). HBV-DNA was quantified by PCR (Cobas Amplicor test; Roche Diagnostic, Basel, CH) and CMV-DNA was quantified with artus-CMV-LC PCR (Qiagen, Qiagen Gmbh, Hilden), EBV-DNA was tested with EBV R-gene DNA extraction and quantification kit (Argene, Varilhes, France). Dengue detection was performed by RNA isolation from serum samples using RNA extraction kit followed by reverse-transcription into cDNA (Superscript III First Strand kit, Invitrogen, California, USA). The cDNA was PCR amplified for detection of the virus, for determination of serotype and for quantification of viral load as previously published [39]. The serum and nasal swab samples were tested for the presence of Influenza A and Adenovirus using RT-PCR (Superscript III First Strand kit, Invitrogen, California, USA) and direct immunofluorescence assay on nasal swabs (Light Diagnostic Influenza A antibody FITC reagent, Millipore, Billerica, MA). Amino acid sequence alignment was done using BLAST from NCBI PubMed (http://blast.ncbi.nlm.nih.gov/Blast.cgi).

### Reagents

HLA-peptide pentameric complexes (pentamers) were purchased from Proimmune (Oxford, UK). Anti-CD8 (PE-Cy7 and APC-Cy7), anti-CD3 (perCP and perCP-Cy5.5) anti-CD38 (APC), anti-HLA-DR (Pe-Cy7), anti-KI-67 (FITC and PE), anti-Bcl-2 (FITC), anti-IFN-γ (FITC and APC), anti-IL2 (FITC, PE and APC), and isotype control antibodies were purchased from BD Biosciences, San Jose, CA.

### Phenotypic analysis

Titrated pentamers (PE) were added to 50 µl of purified PBMC (2×10^6^ cells total) for 15 min at 25°C in the dark, washed and then panel of titrated antibodies for surface markers were added to pentamer stained or total PBMC. The cells were then fixed and permeabilized using Cytofix/Cytoperm solution (BD Biosciences, San Jose, CA). After washing, intracellular staining was performed for intracellular markers (Ki-67, Bcl-2). Cells were then washed 3 times, and fixed with 1% formaldehyde before acquisition on a FACS Canto flow cytometer. Compensation was checked regularly using FASC Diva software. Compensation controls were individually determined for each experimental setup.

### Functional assays

PBMC were stained with the relevant pentamers (MHC/peptide pentamer stimulated), or left unstained (unstimulated), washed and then incubated for 5 h with 10 µg/ml brefeldin A (Sigma-Aldrich, St. Louis, MO). Following incubation, the unstimualted cells were stained with relevant pentamers and MHC/peptide pentamer-stimulated were left in PBS. Then cells were stained with anti-CD8 and anti-CD3 mAbs for 20 min at 4°C then fixed and permeabilized using Cytofix/Cytoperm solution. Finally, cells were stained with anti-IFN-γ and anti-IL-2 for 30 min on ice, washed, and fixed with 1% formaldehyde before acquisition on a FACS Canto flow cytometer. For analysis of anti-virus-specific CD8 T activation *in vitro*, freshly isolated PBMC or purified CD8+ T cells were incubated in vitro at 2×10^6^/ml with or without cytokines (IL-7, IL-2, IL-15, IFN-γ, IFN- α, TNF- α, purchased from RnD Systems, Minneapolis, MN). The cells were collected at indicated time points, and the intracellular cytokine staining was performed as described above.

### Ethics statement

This study was conducted according to the principles expressed in the Declaration of Helsinki. The study was approved by the Institutional Review Board of Singapore National Healthcare Group Ethical Domain and Azienda Ospedaliera Universitaria di Parma Ethical Committee hospitals. All patients provided written informed consent for the collection of samples and subsequent analysis.

## Supporting Information

Figure S1 Different activation markers expression profiles of Influenza, HCMV-specific and HBV-specific CD8+ cells present in a representative patient at the onset of acute hepatitis B.
(1.28 MB TIF)

Figure S2 A) Quantity of CMV, EBV and Flu specific CD8 T cells do not change during heterologous acute viral infections. PBMCs of acute HBV and Influenza patients from three time points of the disease were stained with pentamers specific for CMV, EBV and Flu and with anti-CD3, anti-CD8 monoclonal antibodies. The quantity of pentamer+ cells were determined based on the frequency of pentamer CD8 T cells and the lymphocyte counts. B) Activation of two distinct epitopes of EBV during acute hepatitis B. PBMCs of acute HBV patient were stained with two EBV pentamers (BZLF1 190-197 and EBNA3A 193-201) and CD3, CD8, CD38, HLA-DR surface markers.
(1.26 MB TIF)

Table S1 Pentamers for HBV, EBV, HCMV and Influenza A
(0.03 MB DOC)
