# Supplementary figures and images for: Contribution of Herpesvirus Specific CD8 T Cells to Anti-Viral T Cell Response in Humans
Source: PLoS Pathog. 2010 Aug 19;6(8):e1001051. doi: 10.1371/journal.ppat.1001051 (PMC2924358; doi:10.1371/journal.ppat.1001051)

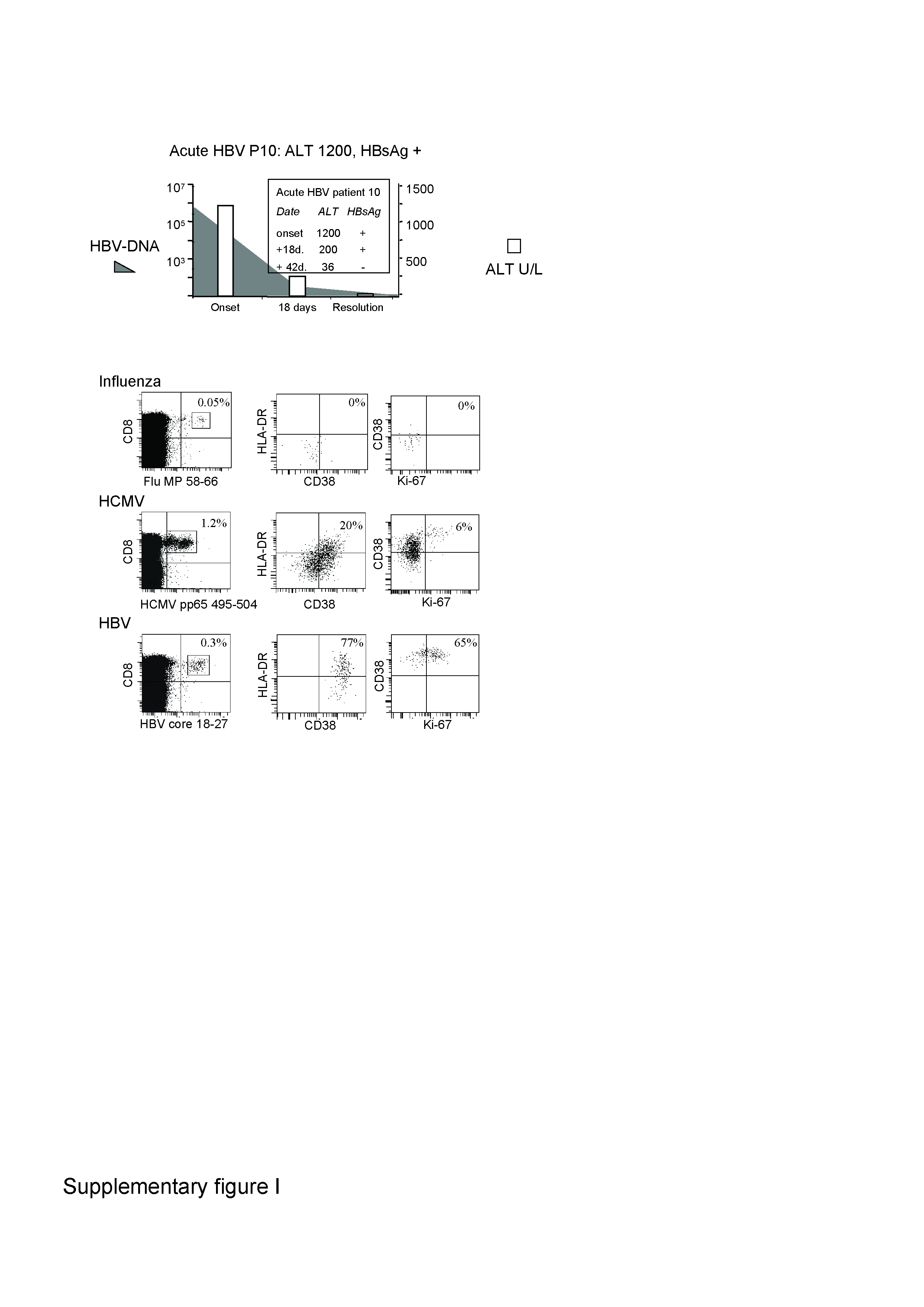

Supplement: Figure S1 — Different activation markers expression profiles of Influenza, HCMV-specific and HBV-specific CD8+ cells present in a representative patient at the onset of acute hepatitis B. (1.28 MB TIF) [file ppat.1001051.s001.tif]

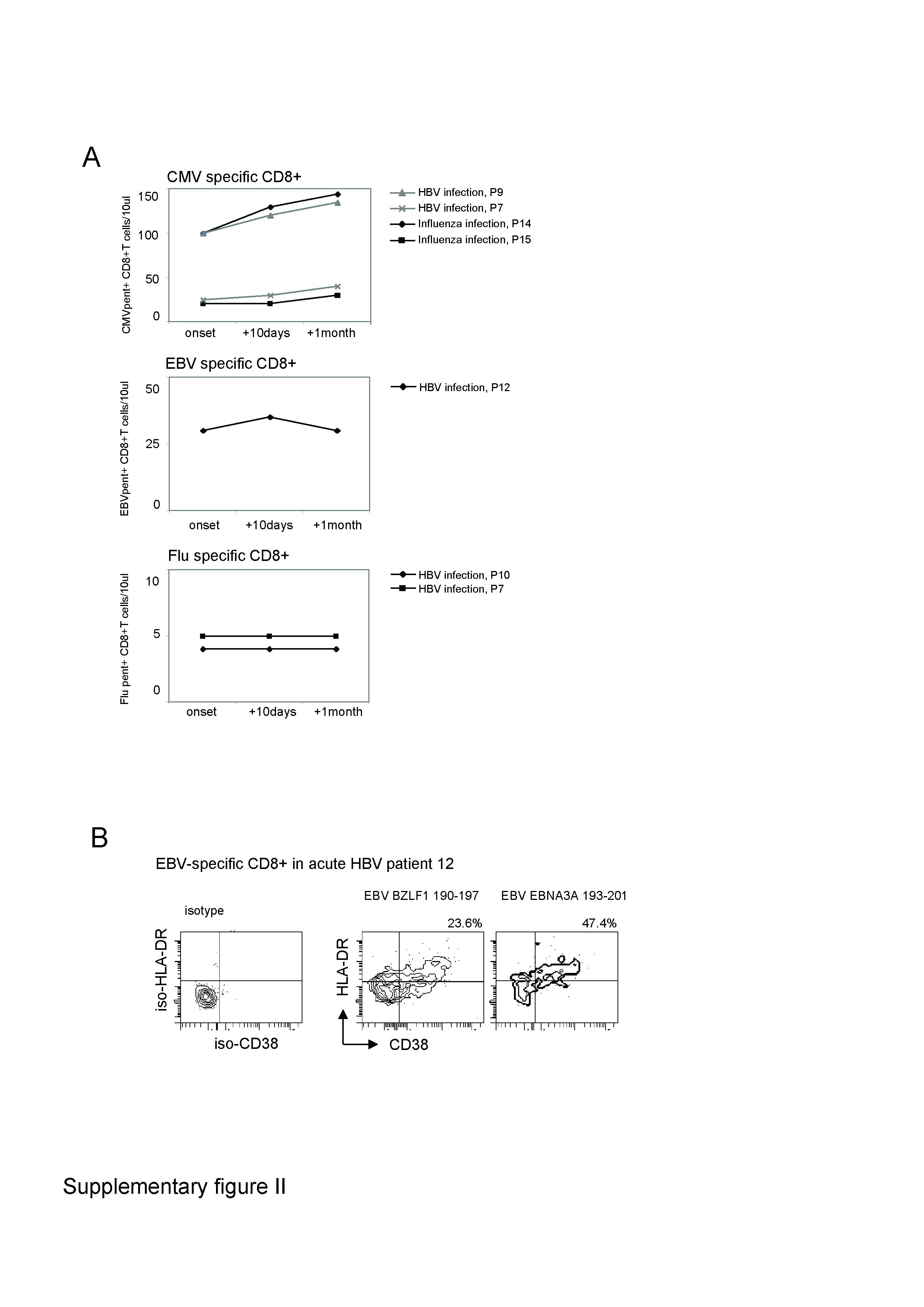

Supplement: Figure S2 — A) Quantity of CMV, EBV and Flu specific CD8 T cells do not change during heterologous acute viral infections. PBMCs of acute HBV and Influenza patients from three time points of the disease were stained with pentamers specific for CMV, EBV and Flu and with anti-CD3, anti-CD8 monoclonal antibodies. The quantity of pentamer+ cells were determined based on the frequency of pentamer CD8 T cells and the lymphocyte counts. B) Activation of two distinct epitopes of EBV during acute hepatitis B. PBMCs of acute HBV patient were stained with two EBV pentamers (BZLF1 190-197 and EBNA3A 193-201) and CD3, CD8, CD38, HLA-DR surface markers. (1.26 MB TIF) [file ppat.1001051.s002.tif]
