# Supplementary material for: Contribution of Herpesvirus Specific CD8 T Cells to Anti-Viral T Cell Response in Humans
Source: PLoS Pathog. 2010 Aug 19;6(8):e1001051. doi: 10.1371/journal.ppat.1001051 (PMC2924358; doi:10.1371/journal.ppat.1001051)
Supplement: Table S1 — Pentamers for HBV, EBV, HCMV and Influenza A (0.03 MB DOC) [file ppat.1001051.s003.doc]

**Online supplemental material**

Supplementary Table 1. Pentamers for HBV, EBV, HCMV and Influenza A

| HLA restriction | **HBV** | **EBV** | **HCMV** | **Influenza A** |
| --- | --- | --- | --- | --- |
| **A0201** | FLLTRILTI, envelope 183-191  FLPSDFFPSV, core18-27  FLLSLGIHL, polymerase 573-581 | CLGGLLTMV, LMP-2 426-434  GLCTLVAML, BMLF-1 259-267  FLYALALLL, LMP-2 356-364 | NLVPMVATV, pp65 495-504 | GILGFVFTL, MP 58-66 |
| **A2402** |  | TYGPVFMSL, LMP2 419–427 | VYALPLKML, pp65 113-121  QYDPVAALF, pp65 341-349 |  |
| **A1101** |  | IVTDFSVIK, EBNA-4 416-424  SSCSSCPLSK, LMP-2 340-349 |  | RMVLASTTAK, MP1 178-187  KSMREEYRK, MP2 70-78 |
| **B0702** |  | RPPIFIRRL, EBNA-3A 247-255 | RPHERNGFTVL, pp65 265-275  TPRVTGGGAM, pp65 417-426 |  |
| **B0801** |  | RAKFKQLL, BZLF-1 190-197  FLRGRAYGL, EBNA-3A 193-201 |  |  |
| **B3501** |  | HPVGEADYFEY, EBNA-1 407-417 | IPSINVHHY, pp65 123-131 |  |
